# Gut metaproteomics reveals activated arginine catabolism and impaired arginine biosynthesis in systemic lupus erythematosus

**DOI:** 10.1128/msystems.00467-26

**Published:** 2026-07-10

**Authors:** Wenjun Xiong, Xuejia Zheng, Ling Yuan, Lianghong Yin, Donge Tang, Yong Dai, Qingwen Wang

**Affiliations:** 1 Clinical Medical Research Center, Shenzhen People's Hospital (The First Affiliated Hospital, Southern University of Science and Technology; The Second Clinical Medical College, Jinan University) 12387, Shenzhen, China; 2 Guangdong Provincial Autoimmune Disease Precision Medicine Engineering Research Center, Shenzhen Autoimmune Disease Engineering Research Center, Shenzhen People’s Hospital (The First Affiliated Hospital, Southern University of Science and Technology; The Second Clinical Medical College, Jinan University), Shenzhen, China; 3 Shenzhen Clinical Research Center for Geriatrics, Shenzhen People’s Hospital (The First Affiliated Hospital, Southern University of Science and Technology; The Second Clinical Medical College, Jinan University), Shenzhen, China; 4 School of Medicine, Anhui University of Science and Technology, Huainan, China; 5 Department of Nephrology, The First Affiliated Hospital of Jinan University, Guangzhou, China; 6 Future Medical Center, Shenzhen University of Advanced Technology 704566, Shenzhen, China; 7 Department of Rheumatism and Immunology, Peking University Shenzhen Hospital 74573, Shenzhen, China; Child Health Research Foundation, Dhaka, Bangladesh

**Keywords:** systemic lupus erythematosus, gut microbiome, metaproteomics, arginine metabolism, functional redundancy

## Abstract

The resilience of microbial metabolic functions during gut microbiome dysbiosis depends on functional redundancy across taxa. However, this ecological principle remains largely unexplored in human autoimmune diseases such as systemic lupus erythematosus (SLE). Here, we utilized quantitative metaproteomics to analyze fecal samples from 103 SLE patients and 62 healthy controls. Analysis of 30,124 protein groups revealed a protein-abundance-based shift in microbial arginine pathway capacity. Specifically, argininosuccinate synthase (ArgG), the committed enzyme for arginine biosynthesis, was significantly downregulated in SLE. In contrast, carbamate kinase and ornithine carbamoyltransferase—key enzymes of the arginine deiminase catabolic pathway—were upregulated. Taxonomic attribution demonstrated that ArgG expression was driven almost exclusively by *Ruminococcus*, a genus heavily depleted in SLE. Conversely, upregulated catabolic enzymes and IMP dehydrogenase (IMPDH, the rate-limiting enzyme in *de novo* purine biosynthesis) were broadly distributed across multiple genera, buffering them against compositional shifts. A leakage-free random forest analysis integrating taxonomic and functional features showed moderate internal discrimination between SLE patients and healthy controls, with a mean area under the curve of 0.784, and identified IMPDH as the most frequently selected functional feature. Because arginine availability regulates T cell function through the GCN2 starvation-response pathway, this protein-abundance-based vulnerability of microbial arginine biosynthesis provides a candidate link between gut dysbiosis and SLE immune pathogenesis.

**IMPORTANCE:**

By applying quantitative metaproteomics to a large clinical cohort, we demonstrate that the metabolic consequences of gut dysbiosis in systemic lupus erythematosus (SLE) are largely dictated by the degree of functional redundancy within the microbiota. We show that functions restricted to a single bacterial lineage, such as *Ruminococcus*-dependent arginine biosynthesis, are highly vulnerable to ecological disruption. Conversely, pathways distributed across diverse taxa—like nucleotide biosynthesis and arginine catabolism—remain robust despite taxonomic shifts. This asymmetric functional distribution shifts the perspective of SLE-associated dysbiosis from broad taxonomic profiling to the precise prediction of metabolic deficits. Crucially, identifying reduced microbial arginine-biosynthetic enzyme abundance provides a candidate microbe-derived link to the arginine-dependent T cell defects characteristic of SLE pathogenesis.

## INTRODUCTION

Systemic lupus erythematosus (SLE) is a chronic multisystem autoimmune disease driven by autoantibody production, immune complex deposition, and progressive organ damage (1). With a global prevalence estimated at 43.7 per 100,000 persons and approximately 3.41 million individuals affected worldwide, predominantly young women of childbearing age, SLE imposes a substantial disease burden (2). Mortality among SLE patients remains elevated compared to the general population despite advances in immunosuppressive therapy (3), underscoring the need for deeper mechanistic understanding and novel therapeutic approaches.

Gut microbiota dysbiosis has emerged as an environmental factor in SLE pathogenesis. The human intestinal tract harbors over 10^14^ microorganisms and contains nearly 70% of total immune cells within gut-associated lymphoid tissues (4). Multiple studies have documented characteristic taxonomic alterations, including a decreased Firmicutes-to-Bacteroidetes ratio, depletion of butyrate-producing commensals such as *Faecalibacterium prausnitzii*, and enrichment of pathobionts including *Ruminococcus gnavus* (5–7). *F. prausnitzii* exerts inflammation-suppressing effects through butyrate production and NF-κB inhibition (8, 9). Its depletion, combined with impaired barrier integrity, may expose microbial components to immune cells and exacerbate systemic autoimmunity (10, 11).

When specific taxa are lost from the SLE gut, the determination of which metabolic functions are impaired and which are preserved requires investigation. The answer depends on how metabolic labor is distributed across the community, a dimension that taxonomic profiling alone cannot resolve (12). Metaproteomics enables direct quantification of expressed microbial proteins, revealing active microbial metabolic processes (13–16). Metaproteomics captures both the identity and the taxonomic origin of actively expressed enzymes, making it possible to assess whether a given metabolic function is sustained by many taxa or depends on one (17). This capacity to link function to taxonomy at the protein level is essential for understanding how compositional shifts translate into functional consequences.

Comprehensive metaproteomic characterizations of the gut microbiota in SLE remain scarce, and one metabolic axis in particular has been studied from the immune end without ever being closed at the microbial end. L-arginine availability has been linked to T cell function for 2 decades. When arginine is scarce, T cells stall at the G0-to-G1 transition through GCN2-mediated sensing of uncharged tRNA, downregulate the CD3ζ chain, and lose responsiveness (18–20). When arginine is abundant, the same cells generate more central memory progeny and mount stronger effector responses (21). These observations come from controlled *in vitro* systems and from mouse models in which arginine concentration could be set by the experimenter. The chain of evidence is tight at the cellular and molecular level. What it does not address is upstream. Where does the luminal arginine that gut-resident T cells encounter actually come from, and is the supply perturbed in patients whose immune system is already deranged? Microbial arginine metabolism is a plausible answer. Gut bacteria run two opposing arms of this pathway. The biosynthetic arm condenses citrulline with aspartate through argininosuccinate synthase (ArgG) and replenishes the arginine pool (22, 23). The catabolic arm, the arginine deiminase (ADI) pathway, degrades arginine to ornithine, carbamoyl phosphate, and ATP through the sequential action of ArcA, ornithine carbamoyltransferase (OTC), and carbamate kinase (ArcC) (24). Whether these two arms are balanced or skewed in the SLE gut, and which taxa carry each arm, has not been measured. If the catabolic arm runs ahead of the biosynthetic arm in patients, the established arginine-T cell axis acquires an upstream driver that lives in the lumen rather than in the immune compartment itself. That is the link this study sets out to test.

This study addresses which microbial metabolic functions persist and which are lost when the gut community shifts in SLE by applying quantitative metaproteomics to fecal samples from 103 patients and 62 healthy controls. The results converge on a single principle. Functions sustained by many taxa are buffered against compositional change; functions that depend on one taxon are not. In the SLE gut, arginine biosynthesis turns out to be the extreme case. It rests almost entirely on *Ruminococcus*, a genus that is significantly depleted in patients, and it collapses while the parallel catabolic and nucleotide-biosynthetic machinery, sourced from multiple genera, is preserved or amplified. This taxonomic asymmetry offers a structural explanation for why the patient gut tilts toward arginine consumption rather than replenishment, and it connects compositional dysbiosis to a metabolic axis with established immunological consequences in T cell biology.

## RESULTS

### Metaproteomic profiling of gut microbiome in systemic lupus erythematosus patients

**Fig 1:**
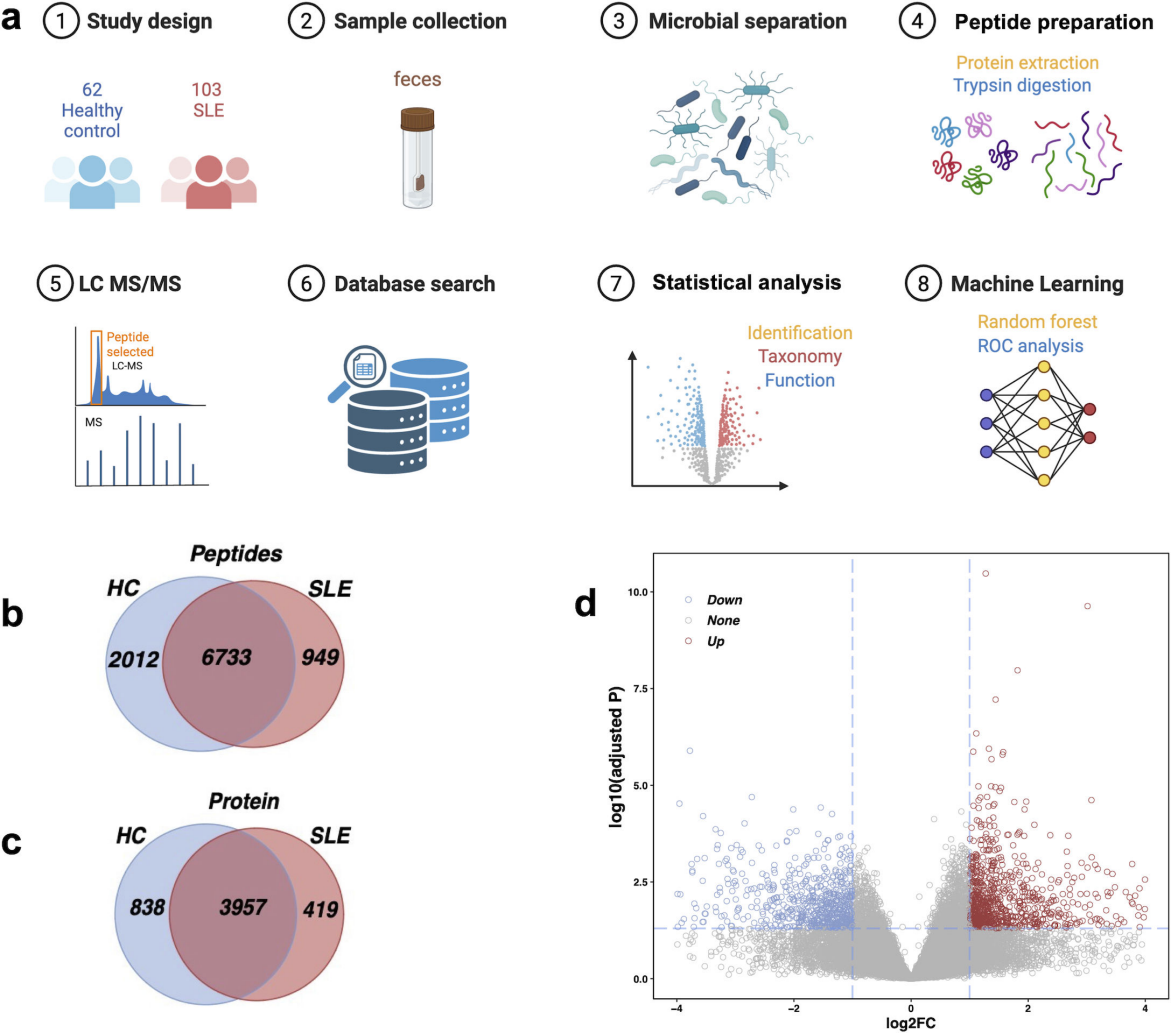
Study workflow and metaproteomic profiling overview. (**a**) Study workflow. Fecal samples from 103 SLE patients and 62 healthy controls (HC) were processed by differential centrifugation for microbial enrichment, followed by protein extraction, trypsin digestion, and label-free DIA mass spectrometry. A spectral library was generated from DDA experiments. (**b**) Venn diagram showing overlap and uniqueness of identified peptides between HC (blue) and SLE (red) groups after filtering (detected in ≥50% of samples per group). (**c**) Venn diagram of identified protein groups between HC and SLE. After filtering, the HC group retained a larger group-level repertoire of detectable peptides and protein groups than the SLE group. (**d**) Volcano plot of differentially abundant proteins (limma with empirical Bayes moderation). Red, significantly upregulated in SLE (log2FC > 1, adjusted *P* < 0.05); blue, significantly downregulated; gray, not significant. A total of 1,534 differentially abundant proteins were identified (853 upregulated, 681 downregulated in SLE).

In this study, we enrolled 103 SLE patients and 62 HC from Peking University Shenzhen Hospital. Clinical characteristics are summarized in Table 1. Fecal samples were collected, and gut microbes were enriched by differential centrifugation (Fig. 1a). After protein extraction and trypsin digestion, label-free data-independent acquisition (DIA) mass spectrometry was performed to identify and quantify microbial proteins using a spectral library generated from data-dependent acquisition (DDA) experiments.

**TABLE 1:** Clinical characteristics at baseline^*a*^

Characteristic	HC (*n* = 62)	SLE (*n* = 103)	*P*-value
Gender (female)	82.26%	97.00%	0.003
Age (years)	33 ± 12	35 ± 10	0.273
BMI (kg/m²)	22.08 ± 4.86	21.60 ± 3.62	0.502
Disease duration (month)	NA	96.53 ± 69.89	–
ESR (mm/h)	NA	15.72 ± 12.07	–
CRP (μg/L)	NA	2.62 ± 4.71	–
IgG (g/L)	NA	12.70 ± 3.34	–
IgM (g/L)	NA	0.99 ± 0.65	–
IgA (g/L)	NA	2.31 ± 0.94	–
CH50 (U/mL)	NA	30.69 ± 9.67	–
C3 (g/L)	NA	0.80 ± 0.22	–
C4 (g/L)	NA	0.15 ± 0.08	–
Anti-dsDNA antibodies (IU/mL)	NA	132.73 ± 149.18	–
Disease activity			
SLEDAI-2K	NA	6 ± 4	–
Severe/moderate/mild/inactive	NA	4/16/34/49	–
LN	NA	28.28%	–
Rash	NA	21.62%	–
GC (mg)	NA	10.61 ± 11.90	–
MMF	NA	37.84%	–
CSA	NA	25.68%	–
HCQ	NA	86.49%	–
MTX	NA	13.51%	–

^*a*^ Patient information for the metaproteomics sequencing cohort. Continuous variables are expressed as mean ± SD. Categorical variables are expressed as *n* (%). *P*-values were calculated using Student's *t*-test for continuous variables and chi-square test for categorical variables. NA indicates not available or not applicable for the HC group; "–" indicates that statistical comparison was not applicable because these data were only available for the SLE group. HC, healthy control; BMI, body mass index; ESR, erythrocyte sedimentation rate; CRP, C-reactive protein; SLEDAI, SLE disease activity index; LN, lupus nephritis; GC, glucocorticoid; MMF, mycophenolate mofetil; CSA, ciclosporin A; HCQ, hydroxychloroquine; MTX, methotrexate.

A total of 30,124 protein groups and 128,420 peptides were identified and quantified across all 165 samples (see Data S1 at https://github.com/WenjunXiongBear/SLE-gut-metaproteomics). On average, 7,562 ± 2,600 protein groups were identified per sample from HC, compared to 7,161 ± 2,833 from SLE patients. Quality control samples were analyzed at regular intervals, with a median coefficient of variation of approximately 40%, within the accepted range for metaproteomic studies given the inherent complexity of gut microbiome samples (Fig. S1).

After filtering for features detected in at least 50% of samples within each group, 9,694 peptides and 5,214 protein groups were retained for comparative analysis. At the peptide level, 6,733 peptides were shared between HC and SLE patients, while 2,012 were unique to HC and 949 unique to SLE patients (Fig. 1b). At the protein level, 3,957 protein groups were shared between groups, with 838 unique to HC and 419 unique to SLE patients (Fig. 1c). After applying the 50% within-group detection filter, the HC group retained a larger group-level repertoire of detectable peptides and protein groups than the SLE group.

Differentially abundant proteins between SLE patients and HC were determined using limma with empirical Bayes moderation (25). Proteins with absolute log2 fold change greater than 1 and adjusted *P* less than 0.05 were considered significantly differential. A total of 1,534 differentially abundant proteins were identified, including 853 upregulated and 681 downregulated in SLE (Fig. 1d; see Data S2 at https://github.com/WenjunXiongBear/SLE-gut-metaproteomics).

### Taxonomic dysbiosis of the gut microbiome in systemic lupus erythematosus characterized by depletion of multiple functional guilds

To characterize gut microbiome alterations in SLE, we profiled the taxonomic composition across all 165 samples using protein-inferred taxonomy (see Materials and Methods). Principal coordinates analysis (PCoA) revealed a statistically detectable but modest group-associated shift in taxonomic composition (PERMANOVA: *P* = 0.004, *R*^2^ = 0.017; Fig. S2a). The low *R*^2^ value reflects the substantial inter-individual variation characteristic of human gut microbiome studies (26). These taxonomic changes were characterized by depletion of several commensal genera. Butyrate-producing genera including *Faecalibacterium* (*P* = 0.0011), *Ruminococcus* (*P* = 0.00048), and [*Eubacterium*] *siraeum* (*P* = 0.015) were significantly depleted in SLE patients, as was *Prevotellamassilia* (*P* = 0.0034; Fig. S2b and c). These findings are consistent with prior 16S rRNA and metagenomic studies of SLE gut microbiota (5–7, 10). Notably, *Mediterraneibacter* (*P* = 0.00078) was the only genus significantly enriched in SLE patients (Fig. S2b). Although its functional contribution to the metabolic alterations described below remains unclear, this selective enrichment indicates that SLE-associated dysbiosis was not limited to depletion of commensal guilds alone.

### Functional characteristics reveal a proliferation metabolic mismatch

**Fig 2:**
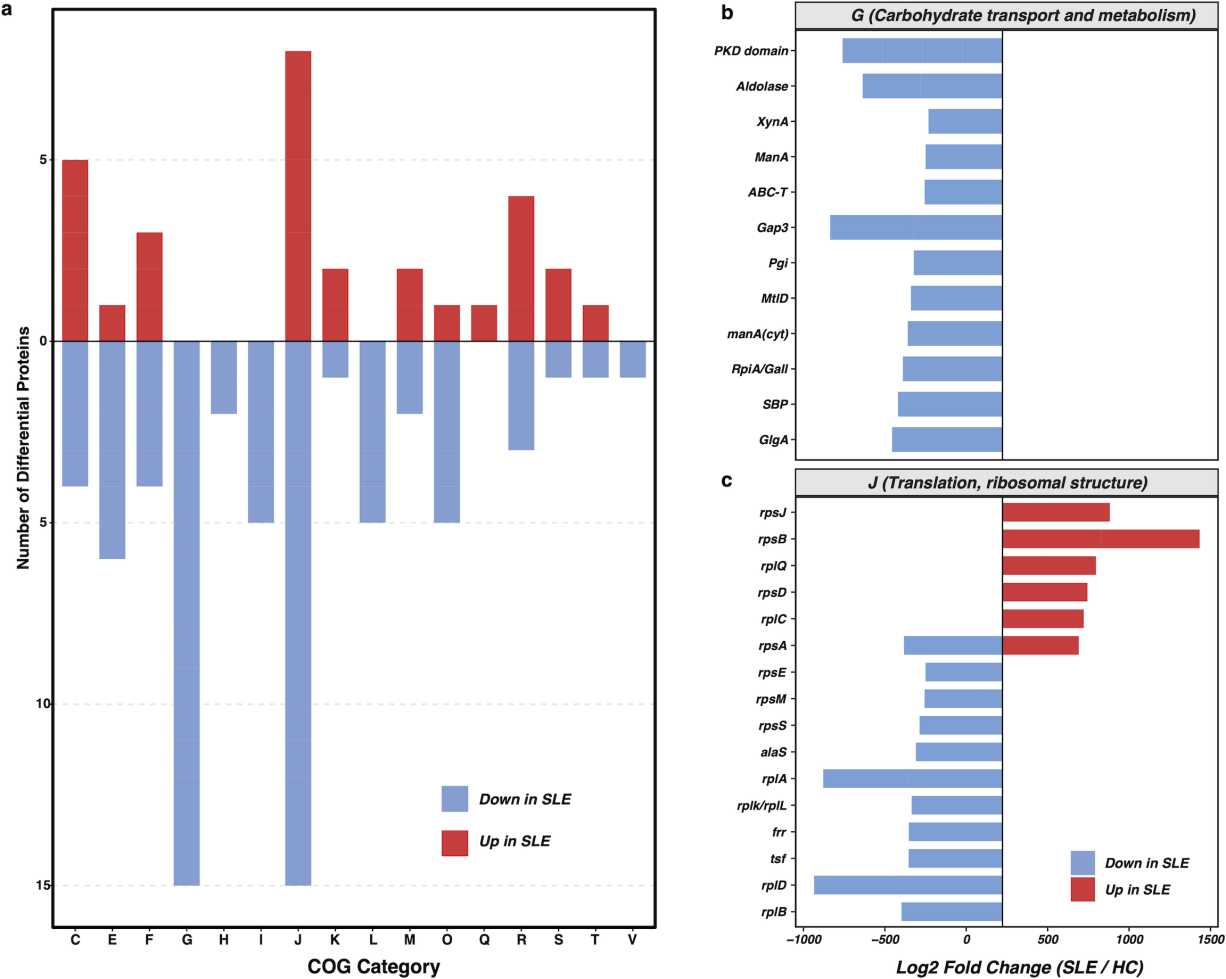
Functional profiling reveals a proliferation-metabolism mismatch in the SLE microbiome. (**a**) Number of differentially abundant proteins per COG functional category. Red bars (above zero line) indicate proteins upregulated in SLE; blue bars (below zero line) indicate downregulation. Category J (translation, ribosomal structure) shows the most differential proteins overall, while category G (carbohydrate transport and metabolism) is predominantly downregulated. (**b**) Log2 fold change of individual differentially abundant proteins within COG category G. All significant proteins in this category are downregulated in SLE, including key glycolytic enzymes (Gap3, Pgi, Aldolase, ManA), consistent with impaired fermentative energy metabolism. (**c**) Log2 fold change of individual differentially abundant proteins within COG category J. A bimodal pattern is evident: a subset of ribosomal proteins (rpsJ, rpsB, rplQ, rpsD, rplC) is upregulated (red), while the majority (rpsA, rpsE, rpsM, rplA, rplD, rplB) is downregulated (blue), suggesting translational stress or selective ribosomal remodeling. A, RNA processing and modification; B, chromatin structure and dynamics; C, energy production and conversion; D, cell cycle control, cell division, and chromosome partitioning; E, amino acid transport and metabolism; F, nucleotide transport and metabolism; G, carbohydrate transport and metabolism; H, coenzyme transport and metabolism; I, lipid transport and metabolism; J, translation, ribosomal structure, and biogenesis; K, transcription; L, replication, recombination, and repair; M, cell wall/membrane/envelope biogenesis; N, cell motility; O, post-translational modification, protein turnover, and chaperones; P, inorganic ion transport and metabolism; Q, secondary metabolite biosynthesis, transport, and catabolism; R, general function prediction only; S, function unknown; T, signal transduction mechanisms; U, intracellular trafficking, secretion, and vesicular transport; V, defense mechanisms; W, extracellular structures; X, mobilome: prophages and transposons; Y, nuclear structure; Z, cytoskeleton.

We investigated the functional state of the microbiome at the proteome level. A broad downregulation of carbohydrate transport and metabolism proteins (COG category G) was evident in SLE (Fig. 2a). Key glycolytic enzymes, including Gap3, Pgi, Aldolase, and ManA, were significantly depleted (Fig. 2b), consistent with impaired energy-yielding metabolism.

Proteins associated with translation and ribosomal structure (COG category J) showed significant enrichment overall, yet the pattern was not uniform. While most ribosomal proteins (rpsA, rpsE, rpsM, rplA, rplD) were downregulated, a distinct subset (rpsJ, rpsB, rpsD, rplC) displayed paradoxical upregulation (Fig. 2c). This bimodal expression pattern may reflect translational stress or selective ribosomal remodeling. These data suggest that the SLE-associated microbiome exhibits reduced fermentative capacity alongside partial enhancement of translational machinery while exhibiting signs of biosynthetic strain.

### Arginine catabolism is activated while biosynthesis is impaired

**Fig 3:**
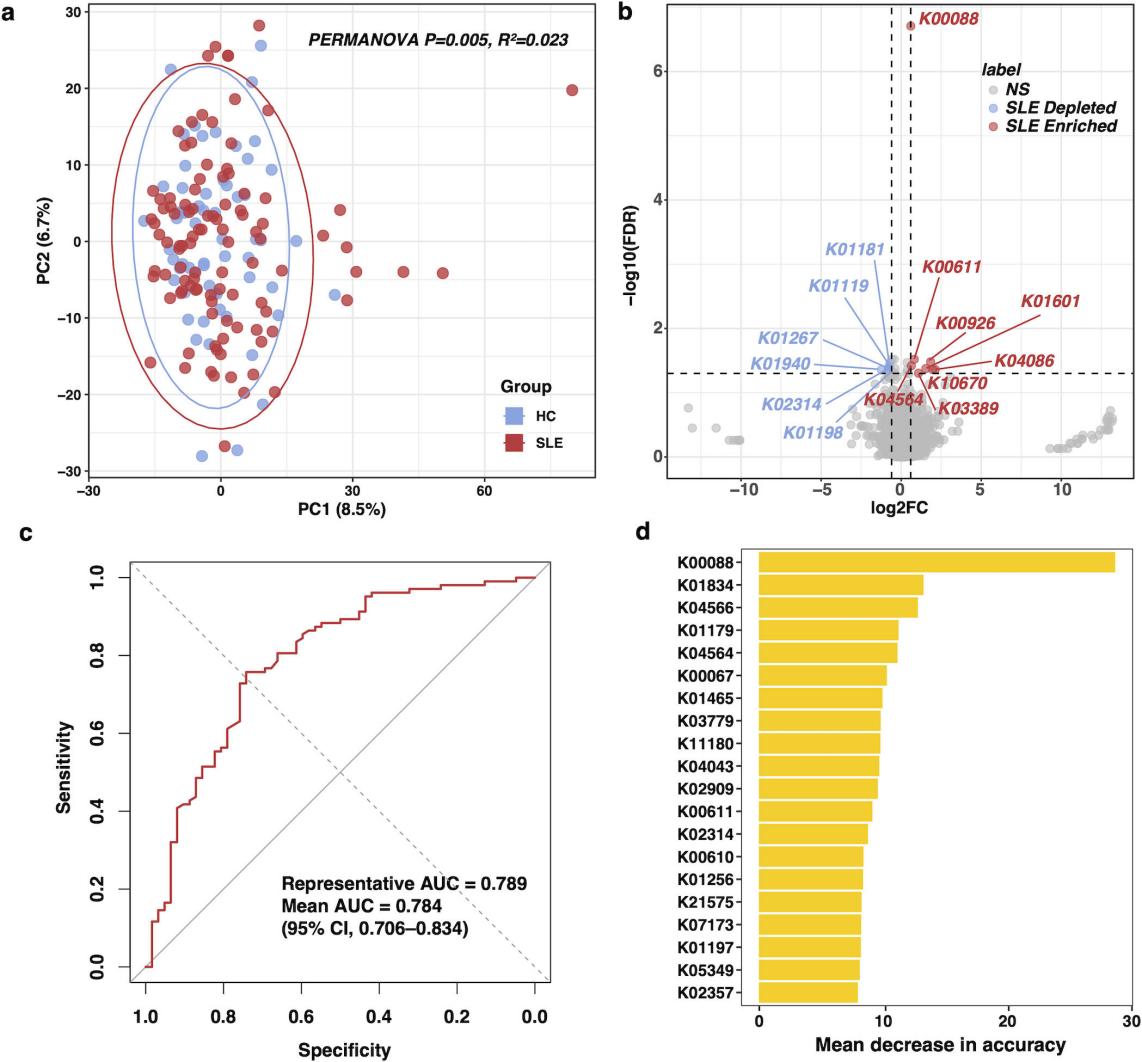
KO-level differential analysis identifies arginine and purine metabolism as selectively altered pathways with moderate classification performance under leakage-free cross-validation. (**a**) Principal component analysis of KO abundance profiles. PC1 and PC2 explain 8.5% and 6.7% of variance, respectively. Ellipses represent 95% confidence intervals. PERMANOVA: *P* = 0.005, *R*² = 0.023. (**b**) Volcano plot of differentially abundant KOs (Wilcoxon rank-sum test, false discovery rate [FDR]-corrected). Red points indicate KOs significantly enriched in SLE; blue points indicate KOs significantly depleted in SLE. The 14 significantly altered KOs are labeled. K00088 (IMP dehydrogenase [IMPDH]) is the most significantly enriched KO. Among the labeled KOs, K00611 (OTC) and K00926 (ArcC) are enriched in SLE, whereas K01940 (ArgG) is depleted, indicating simultaneous activation of arginine catabolism and impairment of arginine biosynthesis. Dashed lines indicate significance thresholds (|log2FC| > 1, FDR < 0.05). (**c**) Representative receiver operating characteristic (ROC) curve from the leakage-free random forest analysis. Feature selection was performed independently within each training fold, and predictions were generated only for held-out samples. The displayed curve corresponds to the repeat whose out-of-fold AUC was closest to the median across 100 independent repeats of stratified fivefold cross-validation. The representative AUC was 0.789, and the mean AUC across all repeats was 0.784 (95% CI, 0.706–0.834). (**d**) Feature selection frequency across 500 training folds from 100 repeats of stratified fivefold cross-validation. Features are ranked by the proportion of training folds in which they were selected. *Ruminococcus*, *Mediterraneibacter*, *Clostridiales bacterium*, *Faecalibacterium*, and IMPDH (K00088) were among the most consistently selected features. Arginine metabolism-related KOs, including OTC (K00611), ArcC (K00926), and ArgG (K01940), were also repeatedly selected.

To identify the specific metabolic pathways underlying this mismatch, we analyzed enzymatic alterations at the KEGG ortholog (KO) level. Principal component analysis of KO abundance profiles revealed a statistically detectable but modest group-associated shift between SLE and HC, with PC1 and PC2 explaining 8.5% and 6.7% of variance, respectively (PERMANOVA: *P* = 0.005, *R*^2^ = 0.023; Fig. 3a). Differential abundance analysis identified 14 significantly altered KOs (Fig. 3b), uncovering a protein-abundance-based shift in microbial arginine pathway capacity.

**Fig 4:**
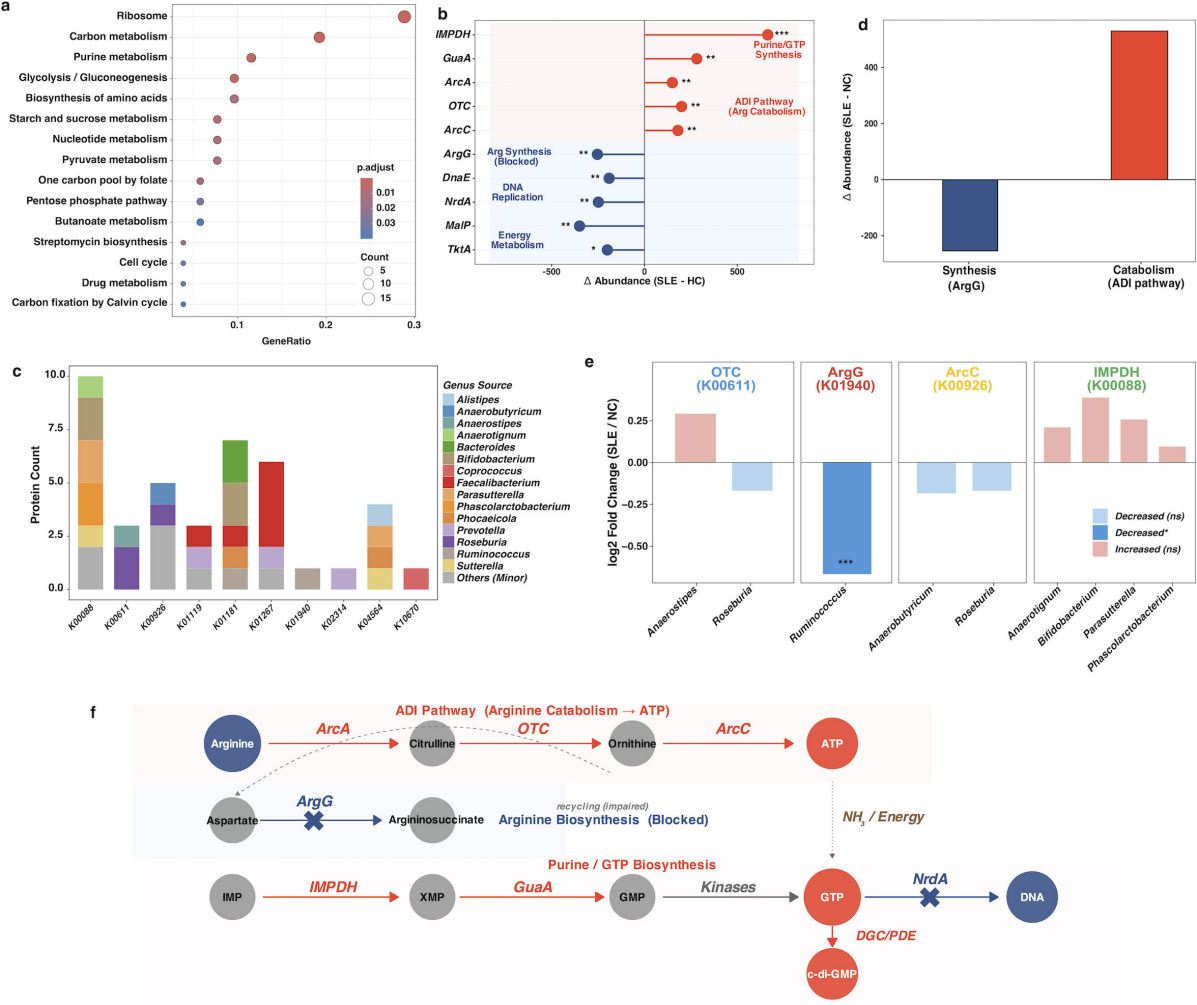
Activated arginine catabolism and biosynthetic impairment driven by asymmetric functional redundancy. (**a**) KEGG pathway enrichment analysis of differentially abundant KOs. Dot size represents gene count; color indicates adjusted *P*-value. Ribosome, carbon metabolism, and purine metabolism rank among the most significantly enriched pathways. (**b**) Lollipop plot of key metabolic drivers grouped by functional category. Background shading distinguishes upregulated functional groups (red: purine/GTP synthesis, ADI pathway) from downregulated groups (blue: arginine synthesis, DNA replication, energy metabolism). Asterisks indicate statistical significance (*P < 0.05, **P < 0.01, ***P < 0.001). (**c**) Taxonomic attribution of differentially abundant KOs. Stacked bars represent the number of proteins contributed by each genus. ArgG (K01940) is assigned predominantly to *Ruminococcus*, while IMPDH (K00088) draws contributions from multiple genera including *Anaerotignum*, *Bifidobacterium*, *Parasutterella*, and *Phascolarctobacterium*. (**d**) Predicted arginine pathway capacity. The left bar shows the abundance change of ArgG, representing reduced biosynthetic enzyme abundance, and the right bar shows the combined abundance change of ADI-pathway enzymes, representing increased catabolic enzyme abundance. The opposing directions indicate a protein-abundance-based shift toward reduced arginine-replenishing capacity and increased catabolic potential. (**e**) Genus-level abundance changes for taxa contributing to OTC, ArgG, ArcC, and IMPDH. Bar color indicates direction and significance of change (dark blue, significantly decreased; light blue, decreased but not significant; light pink, increased but not significant). *Ruminococcus*, the near-exclusive source of ArgG, shows significant depletion (**P < 0.001), whereas taxa contributing to OTC, ArcC, and IMPDH do not show significant abundance changes. (**f**) Integrated metabolic pathway diagram. Upper track: the ADI pathway, in which ArcA, OTC, and ArcC sequentially catabolize arginine to generate ATP. Middle track: ArgG-mediated arginine biosynthetic capacity is impaired (indicated by X). Lower track: IMPDH and GuaA drive purine/GTP biosynthesis. Metabolite nodes are colored by predicted direction of change (red, accumulation; blue, depletion; gray, unchanged). Enzyme labels are colored by expression change (red, upregulated; blue, downregulated; gray, not significant). Dashed arrow indicates impaired recycling of ornithine back to arginine synthesis. Dotted arrow indicates potential flow of NH₃ and energy from ADI pathway to purine biosynthesis.

ArgG (K01940), the enzyme responsible for converting citrulline to argininosuccinate in the arginine biosynthetic pathway, was significantly downregulated in SLE (Fig. 3b; Fig. 4b). Two enzymes of the ADI catabolic pathway were significantly upregulated: OTC (K00611), which converts citrulline to ornithine and carbamoyl phosphate, and ArcC (K00926), which phosphorylates ADP using carbamoyl phosphate to generate ATP (Fig. 3b; Fig. 4b). The simultaneous increase in ADI-pathway enzyme abundance and decrease in ArgG abundance indicates a protein-abundance-based shift toward increased arginine-catabolic potential and reduced biosynthetic replenishment capacity (Fig. 4b and d).

Concurrently, IMPDH (K00088), the rate-limiting enzyme for *de novo* purine biosynthesis, emerged as the most significantly enriched function in the entire data set, accompanied by GuaA (GMP synthase, K01951) (Fig. 3b and 4b). Pathway enrichment analysis confirmed purine metabolism, ribosome, and carbon metabolism among the top altered pathways (Fig. 4). The co-occurrence of enhanced purine synthesis and arginine catabolic activation suggests that energy and nitrogen released through the ADI pathway may fuel nucleotide production and bacterial proliferation.

### Taxonomic attribution reveals single-taxon dependency of ArgG

To assess the taxonomic basis for the observed metabolic asymmetry, we mapped the taxonomic origins of the differentially abundant enzymes governing these interconnected pathways (Fig. 4c). The detectable ArgG signal was assigned almost exclusively to *Ruminococcus* (Fig. 4c and e). This narrow detectable taxonomic sourcing, together with the significant depletion of *Ruminococcus* in SLE (*P* = 0.00048; Fig. S2c), rendered arginine biosynthesis particularly susceptible to compositional shifts. Genus-level analysis confirmed that *Ruminococcus* was the only ArgG-contributing taxon to show a significant abundance decline (Fig. 4e). IMPDH drew contributions from multiple genera, including *Bifidobacterium*, *Phascolarctobacterium*, *Parasutterella*, and *Anaerotignum* (Fig. 4c and e). This distributed taxonomic sourcing may buffer the detectable IMPDH signal against the loss of any individual taxon. ArcC was similarly contributed by multiple genera, specifically *Anaerobutyricum* and *Roseburia*, neither of which showed a significant abundance change in SLE (Fig. 4c and e). OTC occupied an intermediate position, with detectable contributions from both *Anaerostipes* and *Roseburia*, which may explain why the OTC signal was preserved despite depletion of the narrowly sourced ArgG signal (Fig. 4c and e).

The result is a metabolic architecture shaped by the ecological structure of the community (Fig. 4f): ADI-pathway enzyme abundance is increased, whereas ArgG-mediated biosynthetic capacity is impaired. Alternative nitrogen-consuming routes toward nucleotide production remain operational. This pattern, observed for the enzymes examined here, is consistent with a structural consequence of differential functional redundancy across the community. Because sex differed significantly between the SLE and HC groups, we performed a female-only sensitivity analysis including 100 female SLE patients and 51 female healthy controls (Fig. S5). The principal arginine-purine pattern remained consistent in this female-only subset: IMPDH (K00088), ArcC (K00926), and OTC (K00611) remained increased in SLE, whereas ArgG (K01940) remained decreased (Fig. S5a). At the genus level, *Ruminococcus*, the main detectable taxonomic source of ArgG, also remained significantly depleted in female SLE patients (Fig. S5b and c). These findings suggest that the core arginine-purine metabolic pattern was not solely attributable to sex imbalance.

### Diagnostic potential of functional markers

We next evaluated whether functional and taxonomic markers could distinguish SLE patients from healthy controls using random forest classification (27). The integrated model showed moderate internal discrimination, with a mean AUC of 0.784 across 100 repeated cross-validation runs (95% CI, 0.706–0.834; Fig. 3c; Fig. S4a and b). IMPDH (K00088) was the most frequently selected functional feature and was retained in 88.8% of training folds (Fig. 3d). Several taxonomic markers, including *Ruminococcus*, *Mediterraneibacter*, *Clostridiales bacterium*, and *Faecalibacterium*, were also selected with high frequency (Fig. 3d). Arginine metabolism-related KOs, including OTC (K00611), ArcC (K00926), and ArgG (K01940), were repeatedly selected as well (Fig. 3d). Aggregated fold-wise feature importance further supported the contribution of IMPDH and other KO-level features to classification (Fig. S4c). Although based on internal cross-validation, these findings suggest that the arginine-purine functional axis contributes to SLE-associated classification, while its diagnostic performance should be interpreted as moderate and requires external validation in independent cohorts.

## DISCUSSION

### Directional metabolic shifts and their taxonomic basis

Quantitative metaproteomics showed that the gut microbiome of patients with SLE was functionally distinct from that of 62 healthy controls. Differential abundance analysis identified a protein-abundance-based shift in microbial arginine pathway capacity. Two enzymes of the ADI pathway, OTC and ArcC, were significantly upregulated in SLE, whereas ArgG, the committed step in arginine biosynthesis, was markedly downregulated (Fig. 4b and d). IMPDH and GuaA, two enzymes of *de novo* purine biosynthesis, were also enriched, whereas proteins involved in carbohydrate transport and fermentative glycolysis were broadly depleted (Fig. 2a and b). Together, these observations indicate a microbial community with increased abundance of arginine-catabolic and nucleotide-biosynthetic enzymes, alongside reduced abundance of ArgG and broader fermentative energy-metabolism proteins. Maintenance of the ADI pathway despite globally diminished fermentative capacity is consistent with its recognized role as an alternative ATP-generating route in gut anaerobes (24).

The contrasting behavior of ArgG and the catabolic and nucleotide-biosynthetic enzymes appears to reflect the taxonomic breadth of each function. Taxonomic attribution of differentially abundant KOs revealed that the detectable ArgG signal was assigned almost exclusively to *Ruminococcus*, a genus significantly depleted in SLE patients (Fig. 4c and e). In contrast, ArcC, OTC, and IMPDH were contributed by multiple genera whose abundances were not affected to the same extent by dysbiosis. Multi-taxon sourcing likely buffered these functions against compositional perturbation, whereas the near-single-taxon dependency of ArgG left arginine biosynthesis vulnerable to loss of that commensal lineage. This pattern is consistent with the concept of differential functional redundancy in microbial communities (28, 29), which predicts that functions carried by a narrow taxonomic base are structurally exposed during ecological perturbation while multi-taxon functions remain buffered (12, 30). The present data apply this framework to a human disease cohort and identify microbial arginine biosynthesis as a function that occupies the narrow end of the redundancy spectrum in the SLE gut. Whether selective ADI maintenance reflects adaptation to the inflamed gut environment or simply the taxonomic composition that remained after dysbiosis cannot be resolved from cross-sectional protein abundance data alone.

### Relationship to prior SLE microbiome studies

The taxonomic shifts observed here are consistent with the broad pattern reported by earlier 16S rRNA and metagenomic surveys of SLE cohorts. Hevia and colleagues first documented Firmicutes depletion and a reduced Firmicutes-to-Bacteroidetes ratio in Spanish SLE patients (5), and subsequent work in Chinese (6), Korean (31), and additional Chinese cohorts (32) reported a similar pattern across geographically distinct populations, consistently including depletion of butyrate-producing genera such as *Faecalibacterium* and *Ruminococcus*. A recent synthesis confirmed reduced microbial richness and an inverted Firmicutes-to-Bacteroidetes ratio as the most reproducible features of SLE-associated dysbiosis across multiple independent cohorts (7). This protein-level data support these observations and indicate that *Ruminococcus* is depleted at the expression level, not merely at the gene-presence level. By measuring expressed enzymes rather than gene presence, we show that depletion of *Ruminococcus* removes the dominant source of an enzymatic step that no other resident genus reliably performs.

One point of apparent discrepancy with the literature requires clarification. Azzouz and colleagues reported expansion, rather than depletion, of *Ruminococcus gnavus* in lupus nephritis patients, with antibodies against *R. gnavus* cell-wall lipoglycans tracking disease activity (33), and independent reports further linked *R. gnavus glucorhamnan* to TLR-mediated inflammation in inflammatory bowel disease models (34). This does not directly contradict the present finding. Their analysis focused on a single species in a nephritis-enriched cohort, whereas this genus-level attribution captures an aggregate signal across *Ruminococcus*, which encompasses both pathobiont and commensal lineages. Several recent cohorts have reported *Ruminococcus* depletion at the genus level even when *R. gnavus* expansion is detected at the species level (7, 31), indicating that the two findings are reconcilable rather than mutually exclusive. It is biologically plausible that *R. gnavus* expands while other *Ruminococcus* species, including those carrying the ArgG biosynthetic capacity, are simultaneously lost. Resolving this question will require species- or strain-resolved metaproteomics beyond the resolution of the present study. In parallel, the enrichment of *Mediterraneibacter* observed in this cohort warrants further species-level investigation, particularly in light of the taxonomic reassignment of several former *Ruminococcus* lineages and the potential heterogeneity hidden within genus-level labels (35).

The SLE gut microbiome literature has converged on a recurring taxonomic pattern: butyrate producers depleted, certain pathobionts enriched, and the Firmicutes-to-Bacteroidetes ratio inverted (7). The present study does not overturn that pattern. Instead, it reinterprets the existing taxonomic evidence through a functional axis and suggests why one specific axis—arginine metabolism—may be especially vulnerable to the compositional changes already on record.

### Immunological and inflammatory consequences of the metabolic shift

Impaired microbial arginine biosynthesis could have downstream consequences for host immunity. L-arginine availability regulates T cell function through several non-redundant mechanisms. Arginine depletion activates the amino acid stress sensor GCN2, promotes phosphorylation of eukaryotic translation initiation factor 2α, and arrests T cells at the G0-to-G1 transition (19), reduces TCR ζ-chain expression (36, 37), and impairs cyclin D3 and CDK4 induction (19). Restoration of arginine reverses these defects and promotes central memory T cell differentiation with greater effector capacity (21). This arginine-sensing axis also intersects with mTORC1 signaling, which is constitutively activated in SLE T cells and contributes to pathogenic Th17 expansion, impaired Treg function, and the broader metabolic reprogramming that has been directly implicated in lupus pathogenesis (38–40). If the predicted reduction in microbial arginine-replenishing capacity translates into lower luminal arginine availability, the established cellular consequences of arginine restriction may contribute to the T cell dysfunction characteristic of SLE. This remains a hypothesis derived from protein abundance data, and direct testing will require paired metabolomic profiling of fecal and mucosal arginine and citrulline concentrations, ideally across disease flares and remissions.

A second line of interpretation links the enriched nucleotide-biosynthetic machinery to the proliferative state of the community. This interpretation is also consistent with the bimodal ribosomal pattern observed in COG category J, in which a subset of ribosomal proteins increased whereas others decreased, arguing for selective translational remodeling rather than uniform biosynthetic activation. IMPDH and GuaA catalyze rate-limiting steps in *de novo* GTP biosynthesis, and the ATP and ammonia released by ADI-mediated arginine catabolism could feed directly into purine biosynthesis, coupling the two pathways functionally (24). Rapidly proliferating bacteria may release increased quantities of pathogen-associated molecular patterns, including lipopolysaccharide, peptidoglycan, and unmethylated bacterial DNA, which can engage TLR9 on plasmacytoid dendritic cells and B cells (41). TLR9 activation by microbial DNA has been implicated in the type I interferon signature that characterizes SLE pathogenesis (42). More recent syntheses continue to place aberrant nucleic acid sensing and type I interferon amplification at the center of lupus immunopathology, supporting the plausibility of this microbial-to-immune link (43, 44). In addition, IMPDH enrichment may indicate increased microbial guanine nucleotide biosynthetic capacity rather than only a classifier signal. If accompanied by increased purine production or turnover, this shift could theoretically influence host purine availability and uric acid metabolism, although this was not directly measured in the present study (45). Because urate crystals and purine-derived danger signals can activate inflammasome-related inflammatory pathways, the microbial purine axis may represent an additional hypothesis linking dysbiosis to systemic inflammation (46). This interpretation remains speculative and will require paired fecal metabolomics and host inflammatory readouts for validation.

**Fig 5:**
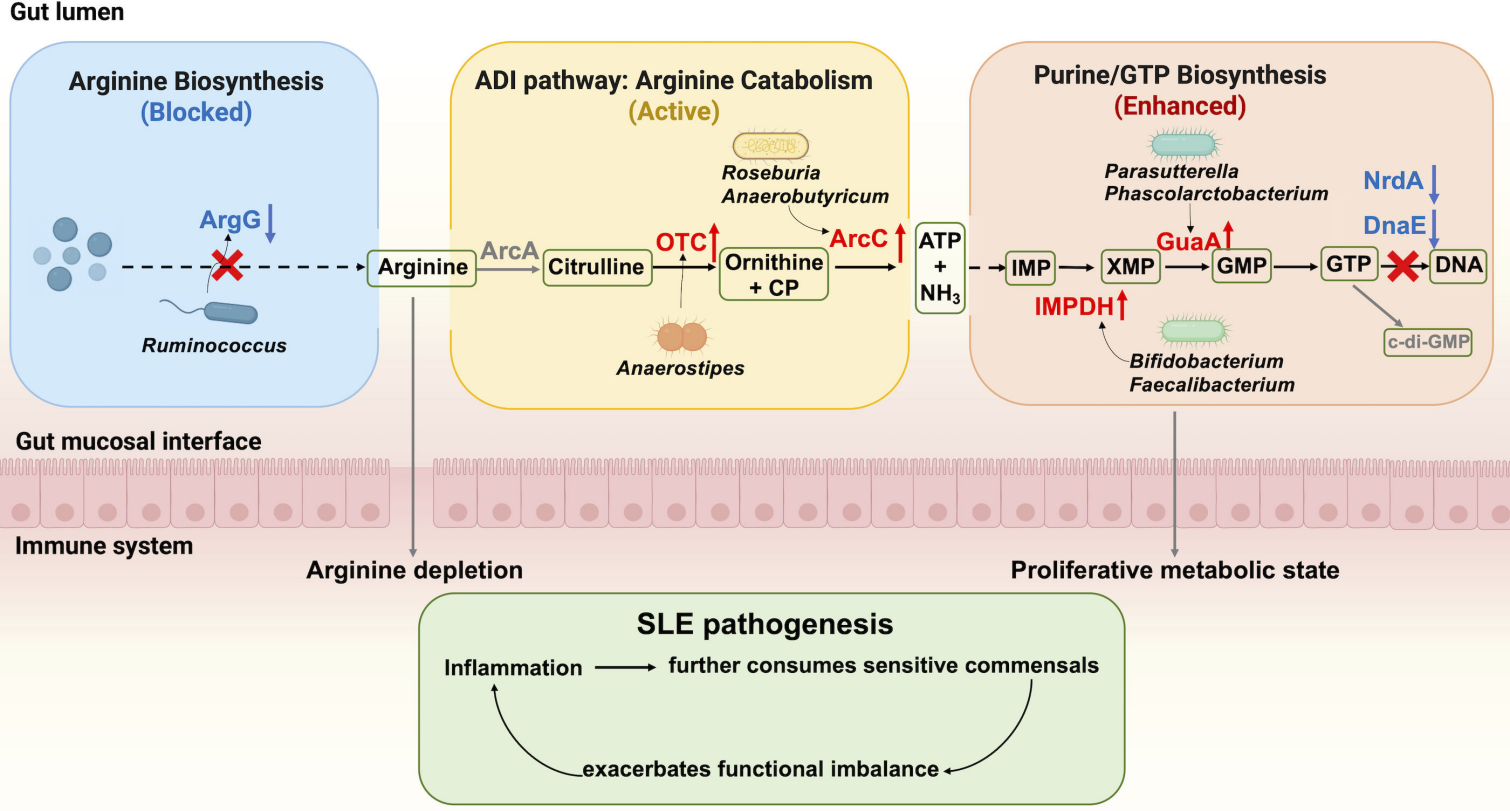
Proposed model linking microbial arginine metabolic disruption to SLE pathogenesis. Schematic illustrating how taxonomic shifts in the SLE gut microbiome are associated with a protein-abundance-based shift in arginine metabolism and enhanced nucleotide-biosynthetic enzyme abundance. The figure is divided into three zones: gut lumen (top), gut mucosal interface (middle), and immune system (bottom). Within the gut lumen, three functional modules are depicted. Left (blue): arginine biosynthesis (impaired). Depletion of *Ruminococcus*, the near-exclusive taxonomic source of argininosuccinate synthase (ArgG), leads to impairment of arginine biosynthetic capacity (indicated by X). Center (yellow): ADI pathway: arginine catabolism (active). Dietary or exogenous arginine is catabolized through the arginine deiminase pathway. ArcA (gray, not statistically significant) converts arginine to citrulline; OTC (red, upregulated) converts citrulline to ornithine and carbamoyl phosphate (CP); ArcC (red, upregulated) generates ATP and NH₃ from CP. Contributing genera (*Anaerostipes* for OTC; *Roseburia* and *Anaerobutyricum* for ArcC) are shown beneath the respective enzymatic steps. Right (pink): purine/GTP biosynthesis (enhanced). IMPDH (red, upregulated) catalyzes IMP to XMP conversion; GuaA (red, upregulated) catalyzes XMP to GMP conversion. GMP is further phosphorylated to GTP. The downstream conversion of GTP to DNA is impaired, as indicated by downregulation of NrdA and DnaE (blue). A gray arrow with a question mark denotes the unconfirmed possibility of GTP diversion toward c-di-GMP. Contributing genera (*Parasutterella*, *Phascolarctobacterium*, *Bifidobacterium*, and *Anaerotignum*) are indicated. Below the gut mucosal interface, two downstream consequences are shown. Reduced arginine-replenishing capacity may contribute to lower arginine availability, which could activate the GCN2 pathway and impair T cell function. The proliferative metabolic state (right) may contribute to immune activation through mechanisms yet to be determined. Both converge on SLE pathogenesis, depicted as a self-reinforcing loop in which inflammation further depletes sensitive commensals and exacerbates the functional imbalance.

These two lines of interpretation converge at the gut mucosal interface, where reduced arginine-replenishing capacity, increased nucleotide-biosynthetic enzyme abundance, and loss of protective commensals may act together. *Faecalibacterium prausnitzii*, a butyrate producer that suppresses NF-κB signaling (8) and supports peripheral Treg induction through butyrate-mediated epigenetic regulation (47), was significantly depleted alongside *Ruminococcus* in this cohort. The combined loss of immunomodulatory metabolites (arginine, butyrate) and gain of inflammatory triggers (PAMPs from proliferating taxa) describes a dual-hit configuration (Fig. 5) that is consistent with the reported association between dysbiosis severity and SLE disease activity in longitudinal clinical studies (7). An exploratory analysis of biofilm-associated functional modules identified c-di-GMP phosphodiesterase as significantly downregulated (Fig. S3), potentially consistent with c-di-GMP accumulation, although no consistent directional enrichment was observed across the four modules examined, precluding firm conclusions on this secondary axis.

### Diagnostic and therapeutic implications

The Random forest analysis should be interpreted cautiously. Under a leakage-free repeated cross-validation framework, the integrated taxonomic and functional model showed moderate internal discrimination rather than strong diagnostic performance (mean AUC, 0.784; 95% CI, 0.706–0.834; Fig. 3c; Fig. S4a and b). IMPDH remained the most influential functional feature and was selected in most training folds, but several taxonomic markers, including *Ruminococcus* and *Faecalibacterium*, were also selected with high frequency (Fig. 3d; Fig. S4c). Thus, the classifier supports the relevance of the arginine-purine axis as a disease-associated signal, but it should not be interpreted as a validated diagnostic model (16, 29). External validation in independent and sex-balanced cohorts will be required before any clinical diagnostic utility can be claimed.

The metabolic signature also overlaps with existing SLE pharmacology. Mycophenolate mofetil (MMF), an IMPDH inhibitor widely used in SLE management, exerts its primary immunosuppressive effect through inhibition of host IMPDH in proliferating lymphocytes (48). Bacterial and human IMPDH share substantial sequence homology, and the most enriched microbial function in this SLE data set maps to the same enzymatic reaction. Whether orally administered MMF reaches luminal concentrations sufficient to inhibit microbial IMPDH activity has not been tested. Gut microbial enzymes are known to metabolize and to be inhibited by orally administered drugs in ways that affect host pharmacokinetics and treatment response (49), placing this question within the broader framework of pharmacomicrobiomics. From a complementary perspective, targeted restoration of arginine biosynthetic capacity—through supplementation with ArgG-competent strains, dietary arginine, or prebiotics favoring *Ruminococcus*—represents a testable hypothesis for future intervention studies. These strategies remain speculative until the predicted metabolite-level depletion is independently confirmed.

### Strengths, limitations, and conclusions

A principal strength of this study is methodological. Quantitative metaproteomics across 165 samples allowed us to measure expressed enzymes and assign them to taxonomic source within the same data set, a combination that 16S rRNA and shotgun metagenomic surveys cannot provide (15). This direct linkage of function to taxonomy at the protein level enabled the analysis of asymmetric functional redundancy that anchors the central conclusion. The cohort size (103 SLE patients and 62 healthy controls) also provided substantial depth for protein-level comparisons between patients and controls.

Several limitations bound the interpretation. The cross-sectional design cannot establish whether the observed metabolic phenotype contributes to disease pathogenesis or instead emerges as a consequence of disease activity; longitudinal sampling across flares and remissions will be required to disentangle cause from consequence. Metaproteomics measures protein abundance rather than metabolite flux, and the predicted arginine-depleting capacity therefore requires confirmation by targeted metabolomic profiling of arginine, citrulline, and purine intermediates. The cohort showed a significant sex imbalance between SLE patients and healthy controls, which may have influenced microbiome-associated comparisons. Although the female-only sensitivity analysis preserved the main arginine-purine findings (Fig. S5), residual sex-related confounding cannot be fully excluded, and validation in sex-balanced cohorts is needed. The cohort also lacks detailed stratification by SLEDAI score and medication regimen, and immunosuppressive agents—particularly glucocorticoids and MMF—may have influenced gut microbiome composition and therefore acted as confounders (33, 49). Protein-inferred taxonomy has lower phylogenetic resolution than DNA-based approaches, and this intensity-weighted attribution method may overrepresent contributions from taxa with high per-cell expression (50). The asymmetric redundancy analysis was restricted to enzymes of the arginine-purine axis; systematic assessment of taxonomic breadth across all detected KOs will be required to establish the generality of this framework in the SLE gut.

In conclusion, the present study identifies a directional imbalance in microbial arginine metabolism in the SLE gut microbiome, characterized by increased catabolism, impaired biosynthesis, and enhanced *de novo* purine synthesis. The biosynthetic impairment is structurally explained by near-single-taxon dependency of ArgG on *Ruminococcus*, a genus consistently depleted in SLE, whereas catabolic and nucleotide-biosynthetic functions are sustained by multi-taxon redundancy. This taxonomic architecture provides a candidate structural link between the gut dysbiosis already documented in SLE and the immune dysregulation that defines the disease, grounded in the established immunological consequences of arginine restriction in T cells. Independent cohort replication, longitudinal sampling, and targeted metabolomic validation are the essential next steps.

## MATERIALS AND METHODS

### Clinical sample collection

A total of 103 patients with SLE and 62 HC were recruited from the Department of Rheumatology and Immunology at Peking University Shenzhen Hospital (Shenzhen, China). All SLE patients met the revised American College of Rheumatology classification criteria. Disease activity was assessed at enrollment using the SLEDAI-2K index. Participants were excluded if they had received antibiotic treatment within 3 months prior to sampling, had co-existing acute or chronic inflammatory diseases, infectious diseases, or malignancies, or had other autoimmune disorders. Clinical data, including demographic characteristics, disease duration, current medications, and laboratory parameters (ESR, CRP, immunoglobulins, complement levels, and anti-dsDNA), were collected within 1 week of fecal sampling. Detailed clinical characteristics, including SLEDAI-2K distribution and medication categories, are summarized in Table 1. Fecal samples were collected in sterile tubes containing OMNIgene-GUT preservation medium (OM-200, DNA Genotek, Ontario, Canada) and stored at −80°C until processing.

### Sample preparation and protein extraction

Microbial proteins were extracted using an optimized SDS-based protocol. Fecal samples were resuspended in SDS lysis buffer supplemented with EDTA and a protease inhibitor cocktail. The mixture was homogenized using a tissue grinder (60 Hz, 2 min) with steel beads. Following centrifugation at 25,000 × *g* for 15 min at 4°C, the supernatant was collected. Proteins were reduced with 10 mM dithiothreitol at 56°C for 1 h and alkylated with 55 mM iodoacetamide in the dark for 45 min at room temperature. Proteins were precipitated using cold acetone (1:5, vol/vol) at −20°C for 30 min. Protein pellets were washed, resolubilized in SDS-free buffer, and quantified using the Bradford assay with bovine serum albumin as the standard. Protein integrity was verified by 12% SDS-PAGE. For enzymatic digestion, 100 μg of protein per sample was incubated with trypsin at a protein-to-enzyme ratio of 40:1 (wt/wt) at 37°C for 4 h. The resulting peptides were desalted using Strata-X columns and vacuum-dried.

### Liquid chromatography-tandem mass spectrometry

To generate a comprehensive spectral library, pooled peptides were fractionated using high-pH reversed-phase chromatography on a Shimadzu LC-20AD system equipped with a Gemini C18 column (5 μm, 4.6 × 250 mm). A linear gradient (5%–95% B, pH 9.8) was applied, and fractions were consolidated into 10 pools. Mass spectrometric analysis was performed on a timsTOF Pro mass spectrometer (Bruker Daltonics, Bremen, Germany) coupled to a nanoElute liquid chromatography system (Bruker Daltonics). Peptides were separated on a reversed-phase C18 column (1.9 μm particle size, 75 μm inner diameter × 25 cm length, IonOpticks, Australia) using a 60 min gradient from 2% to 80% acetonitrile in 0.1% formic acid at a flow rate of 300 nL/min. The column oven temperature was maintained at 50°C. For DDA-based library generation, the instrument was operated in PASEF mode. The ion mobility range was set from 1/K_0_ = 0.6 to 1.6 Vs/cm^2^, and 10 PASEF MS/MS scans were acquired per cycle with a total cycle time of 1.17 s. Precursor ions in the *m*/*z* range of 100–1,700 with charge states 0–5 were selected for fragmentation, and dynamic exclusion was applied for 0.4 min. For DIA-based quantification, the instrument was operated in diaPASEF mode. The ion mobility range (1/K_0_ = 0.6–1.6 Vs/cm^2^) was covered using 16 diaPASEF scans per cycle with variable isolation window widths ranging from 25 to 50 Da across *m*/*z* 400–1,200, resulting in a total cycle time of approximately 1.8 s.

### Proteomic data processing and bioinformatics

Raw DDA data were processed using X!Tandem (version Alanine 2017.2.1.2) and MaxQuant (version 1.5.3.30) to construct the spectral library (51). The Andromeda search engine was employed with the following parameters: trypsin digestion (maximum one missed cleavage), peptide mass tolerance of 10 ppm, and fragment mass tolerance of 0.05 Da. FDR was controlled at 1% at both PSM and protein levels. DIA data were analyzed using MSstats for protein quantification and statistical inference (52). Functional annotation was performed using Diamond (E-value < 1 × 10^−5^) against public databases including NCBI NR, eggNOG v.5.0, and KEGG (53, 54). Taxonomic classification was conducted using MEGAN (version 4.6) based on the lowest common ancestor (LCA) algorithm applied to BLAST results against the NCBI NR database (55).

### Taxonomic attribution of functional annotations

To determine the genus-level taxonomic source of each differentially abundant KO, we combined KEGG functional annotation and LCA-based taxonomic assignment for each identified protein. For a given KO, all proteins assigned to that KO were grouped, and the taxonomic origin of each protein was recorded at the genus level. The contribution of each genus to a given KO was then calculated as the sum of protein intensities from that genus divided by the total protein intensity for that KO across all samples. This intensity-weighted approach captures both the number of contributing proteins and their relative abundance but may overestimate contributions from taxa with high per-cell expression. Results were visualized as stacked bar plots of protein counts per genus for each KO (Fig. 4c) and as genus-level fold change plots (Fig. 4e). For genus-level attribution, proteins assigned only to high-level or ambiguous taxonomic labels were not interpreted as genus-resolved contributors. Therefore, the apparent single-genus dependency of ArgG should be interpreted as the dominant detectable genus-level attribution under this protein-based LCA framework, rather than definitive evidence that ArgG is exclusively encoded or expressed by a single taxon.

### Statistical analysis and machine learning

All statistical analyses were performed in R (version 4.4). Beta diversity was assessed via PCoA based on Bray-Curtis dissimilarity and tested by PERMANOVA (999 permutations). Differential abundance of genus-level and KO-level features was evaluated using the Wilcoxon rank-sum test; protein-level differences were assessed using the limma package (25). *P*-values were adjusted for multiple testing using the Benjamini-Hochberg method where applicable (FDR < 0.05). Random forest classification was performed using the randomForest package (ntree = 1,000; mtry = √*p*, where *p* is the number of features) (27). To avoid information leakage, feature selection was performed independently within each training fold. Specifically, within each training split, genera were ranked by Wilcoxon rank-sum test, and the top 6 differentially abundant genera were retained. KO features were also tested within the training split, and KOs passing the predefined differential-abundance threshold were retained. The selected features from the training split were then used to fit the random forest model, and class probabilities were predicted only for the held-out fold. This procedure was repeated across 100 independent repeats of stratified fivefold cross-validation. Out-of-fold predictions from the fivefolds within each repeat were concatenated to compute one AUC per repeat using the pROC package (56). We report the mean AUC and the 2.5th and 97.5th percentiles of the repeat-level AUC distribution. Feature selection frequency was summarized across all 500 training folds. Feature importance was assessed as the mean decrease in accuracy and summarized across training folds rather than from a model trained on the full data set.

## Data Availability

Analysis code and processed data tables, including Data S1 and Data S2, are publicly available at GitHub: https://github.com/WenjunXiongBear/SLE-gut-metaproteomics.The mass spectrometry proteomics data have been deposited in the MassIVE repository under data set identifier MSV000101802 and doi:10.25345/C59P2WM2R and are available at https://massive.ucsd.edu/ProteoSAFe/dataset.jsp?accession=MSV000101802.

## SUPPLEMENTAL MATERIAL

Supplemental figures msystems.00467-26-s0001.docx Figures S1 to S5.

ASM does not own the copyrights to Supplemental Material that may be linked to, or accessed through, an article. The authors have granted ASM a non-exclusive, world-wide license to publish the Supplemental Material files. Please contact the corresponding author directly for reuse.
