# Supplementary material for: Gut metaproteomics reveals activated arginine catabolism and impaired arginine biosynthesis in systemic lupus erythematosus
Source: mSystems. 2026 Jul 10;11(8):e00467-26. doi: 10.1128/msystems.00467-26 (PMC13483364; doi:10.1128/msystems.00467-26)
Supplement: Supplementary file 1 [file msystems.00467-26-s0001.docx]

**Fig S1. Quality control assessment of metaproteomic analysis.**

Boxplot showing the coefficient of variation (CV) of protein intensities across samples in healthy controls (HC), quality control samples (QC), and SLE patients. The QC samples, consisting of pooled mixtures analyzed at regular intervals throughout the experiment, showed a median CV of approximately 40%, demonstrating acceptable technical reproducibility. The higher CV observed in HC and SLE groups reflects biological heterogeneity among individual subjects rather than technical variation.

**Fig S2. Gut microbiota compositional differences between SLE patients and healthy controls**

**(a)** Principal coordinate analysis (PCoA) based on Bray-Curtis dissimilarity showing a statistically detectable but modest group-associated shift in taxonomic composition between SLE patients (red, n = 103) and healthy controls (HC, blue, n = 62). Ellipses represent 95% confidence intervals. Statistical significance was assessed by PERMANOVA (P = 0.004, R² = 0.017). **(b)** Log2 fold change of differentially abundant genera identified between groups. Blue bars indicate genera enriched in HC; red bars indicate genera enriched in SLE. **(c)** Relative abundance distribution of six significantly altered genera. Each dot represents an individual sample. Box plots display median values with interquartile ranges; whiskers extend to 1.5× IQR. Statistical comparisons were performed using Wilcoxon rank-sum test; p-values are shown for each comparison.

**FigS3. Exploratory analysis of biofilm-associated functional modules**

**(a)** Number of candidate proteins identified for each biofilm-related functional module (c-di-GMP signaling, exopolysaccharide/biofilm, adhesion, motility). **(b)** Volcano plot of differentially abundant biofilm module proteins. Points are colored by module membership; dashed line indicates *P* = 0.05. **(c)** Proportion of upregulated versus downregulated proteins within each module. No module shows a consistent directional shift (all approximately 50/50), indicating that the SLE microbiome does not exhibit a coherent biofilm-like phenotype at the module level. **(d)** Boxplot comparison of total module protein intensity between HC and SLE. Only the c-di-GMP module shows a statistically significant difference (**P < 0.01); biofilm EPS, adhesion, and motility modules are not significantly different (ns).

**Fig. S4. Leakage-free Random Forest classification analysis.**

(a) Distribution of AUC values across 100 independent repeats of stratified five-fold cross-validation. Feature selection was performed independently within each training fold.

(b) Out-of-fold predicted probabilities from the representative repeat, defined as the repeat whose AUC was closest to the median AUC across all repeats.

(c) Aggregated fold-wise Random Forest feature importance, summarized as mean decrease in accuracy across training folds. This analysis supported the contribution of KO-level functional features, including IMPDH (K00088), to the classification model.

**Fig. S5. Female-only sensitivity analysis of the arginine–purine axis.**

(a) Female-only differential abundance results for key KO features. IMPDH (K00088), ArcC (K00926), and OTC (K00611) remained increased in SLE, whereas ArgG (K01940) remained decreased.

(b) Female-only differential abundance results for key genera related to the arginine–purine axis. *Ruminococcus*, the main detectable taxonomic source of ArgG, remained depleted in female SLE patients.

(c) Female-only abundance changes of enzyme-contributing genera. *Ruminococcus*, the main detectable taxonomic source of ArgG, remained significantly depleted, whereas most genera contributing to IMPDH, ArcC, and OTC did not show a consistent significant decrease.
